# Clinical significance of concomitant pectus deformity and adolescent idiopathic scoliosis: systematic review with best evidence synthesis

**DOI:** 10.1016/j.xnsj.2022.100140

**Published:** 2022-06-25

**Authors:** Laurian J.M. van Es, Barend J. van Royen, Matthijs W.N. Oomen

**Affiliations:** 1 Department of Orthopaedic Surgery, Noordwest Ziekenhuis, Wilhelminalaan 12 1815 JD Alkmaar, The Netherlands; 2 Department of Orthopedic Surgery, Amsterdam UMC, University of Amsterdam and Vrije Universiteit, Amsterdam Movement Sciences, Meibergdreef 9, 1105 AZ, Amsterdam, the Netherlands; 3 Department of Pediatric surgery, Amsterdam UMC, University of Amsterdam, Amsterdam Movement Sciences, Meibergdreef 9, 1105 AZ, Amsterdam, the Netherlands; 4 Emma Children's Hospital, Amsterdam UMC, University of Amsterdam, Meibergdreef 9, 1105 AZ, Amsterdam, the Netherlands

**Keywords:** Chest wall deformities, Pectus excavatum, Funnel chest, Pectus carinatum, Pigeon breast, Scoliosis, Adolescent idiopathic scoliosis (AIS), AIS, Adolescent Idiopathic Scoliosis, BES, Best Evidence Synthesis, BMI, Body Mass Index, CA, Cobb Angle, CT, Computed Tomography, HI, Haller Index, PC, Pectus Carinatum, PD, Pectus Deformity, PE, Pectus Excavatum, STA, Sternal Tilt Angle

## Abstract

**Background:**

A misbalance in forces is proposed for causing adolescent idiopathic scoliosis (AIS). AIS is therefore correlated to adjacent musculoskeletal pathologies. Its concomitance with idiopathic pectus deformities (PD) is underexposed. This systematic review analyzes the clinical significance and predictive factors of PD-associated AIS.

**Methods:**

A search was performed in PubMed, UpToDate, Embase, and Cochrane. A study was included if it: assessed the association between PD and scoliosis (category I), reported a prevalence of scoliosis in PD patients (category II), or addressed other topics about PD-associated AIS (category III). Studies in category I discussing predictive factors were appraised using the Quality in Prognosis Studies tool. Because of heterogeneity among the studies, predictive factors were analyzed according to a best evidence synthesis. A mean prevalence of scoliosis in PD patients was calculated using category I and II. Category III was narratively reviewed.

**Results:**

Forty-eight studies were included (I:19, II:21, III:8). Category I comprised 512 patients with PD-concomitant scoliosis. Thirteen studies reported predictive factors, of which 15 concerned the prevalence of scoliosis in PD patients and 12 Cobb Angle (CA) change after PD correction. Compared with AIS, PD seems to develop earlier in adolescence, and PD with concomitant AIS was more frequently reported in older patients. Evidence remained conflicting regarding the association between the severity of PD and that of scoliosis. As opposed to at a younger age, late PD correction is not associated with a postoperative increase of CA. Limited evidence showed that patients with a high CA undergoing PD correction do not experience an increase in CA, though, strong evidence indicated that it would not lead to a decrease in CA. The mean probable prevalence of AIS in PD patients was 13.1%.

**Conclusion:**

Current literature confirms the association between PD and AIS in patients with an indication for PD correction.

**Level of evidence:** III

## Introduction

*Scoliosis* is a 3-dimensional (3D) deformation of the spine, defined by a Cobb angle (CA) of >10°. Adolescent idiopathic scoliosis (AIS) is applied when no cause is found. With a prevalence of 2%-3% in the general population, almost 10% needs brace treatment and 0.1%-0.3% surgical correction [1]. Multiple physical mechanisms explaining a misbalance in forces have been proposed as the cause of AIS [2], correlating AIS to adjacent musculoskeletal pathologies; both cranially [3], caudally [4] and ventrally [5].

*Pectus deformities* (PDs) concern the anterior chest wall and develop in the adolescent growth spurt. The 2 most common types are pectus excavatum (PE, >90% of cases) and pectus carinatum (PC). PD can be idiopathic or syndromic, but its etiology is unclear [6]. The Haller index (HI) describes the severity of PE and is calculated using computed tomography (CT) by dividing the transverse diameter by the shortest anterior-posterior distance of the chest [7]. The HI helps indicating surgical correction [7], mostly applying the Nuss procedure wherein semi-circular metal bars are inserted thorascopically to prop up the sternum [8].

Concomitance of idiopathic PD and AIS was first described in 1989 by Waters et al. [5]. Of 596 children with PD 21.5% had concomitant scoliosis. AIS was more severe, requiring surgical correction more often. Thereafter, the reported prevalence differ widely, the surgical treatment of one deformity is assumed to affect the other and the management of PD with concomitant scoliosis is little evaluated [9,10]. Nonetheless, current assumptions are frequently still based on Waters et al.

This systematic review aims to assess the literature on existing clinical relevance for the association between idiopathic PD and AIS by exploring studies that I) primarily concerned the association; II) reported a prevalence of scoliosis in PD patients; and III) addressed PD-associated AIS in another way.

## Methods

This review is an evidence assessment of PD-associated AIS and its predictive factors. It also serves as a scoping review combining current knowledge about concomitant PD and AIS.

### Search strategy

The Preferred Reporting Items for Systematic Reviews and Meta-Analysis (PRISMA) guidelines were used for this systematic review [11]. One reviewer (LE) searched Cochrane, Embase, PubMed, and UpToDate on December 12, 2021. Broad terms were used as keywords: ‘pectus excavatum’, ‘funnel chest’, ‘pectus carinatum’, ‘pigeon breast’, and ‘scoliosis’ because searching ‘idiopathic scoliosis’ proved to be too narrow. The search was limited for conference abstracts and ongoing trials. The reference lists of fully read articles were checked for additional studies.

### Inclusion criteria

The following 3 categories were constructed as framework for this review. Studies were included if it fit into the criteria of at least one.

Category I: comprising patients with *both* PD and scoliosis, with the association (related or unrelated to treatment) as the primary outcome. Case reports included.

Category II: comprising patients with *either* PD or scoliosis, which reported a prevalence of scoliosis or PD.

Category III: regarding a population with *both* PD and AIS that addressed other relevant topics, e.g. genetics and etiology. Case reports included.

### Exclusion criteria

Excluded were: 1) studies not written in English, Spanish, German, or Dutch; 2) abstracts without full text; 3) reviews; 4) entire syndromic populations; and 5) studies before 1990, since we aimed to update Waters’ review.

### Study selection

One reviewer (LE) performed the screening, full-text reading, and inclusion. Indecisiveness about final inclusion was discussed with a second reviewer (BR).

### Data extraction

Authors, publication year, study type, design, period and purpose, number of patients, population characteristics (primary deformation, gender, age, and inclusion of syndromic disorders), family history concerning PD and AIS, definitions and diagnostic measures of PD and scoliosis, and conclusion regarding the association were extracted from all studies. Additionally, the following information was obtained:

Category I: treatment regarding PD and scoliosis, follow-up, prevalence of concomitant thoracic deformity and predictive factors (if reported), mean HI and CA before (and/or after) treatment, and predictive factors of treatment outcome (if reported).

Category II: prevalence of concomitant thoracic deformity and mean CA.

Category III: relevant results.

### Definitions

PC, PE, and scoliosis were defined as by the original authors. Syndromic disorders were defined as genetic, chromosomal, or congenital malformations. Other concomitant diseases were omitted. In this review, the term ‘AIS’ was used only when populations were confirmed idiopathic. The term ‘scoliosis’ was used in all remaining situations.

### Methodological quality

#### Category I

Methodological appraisal was performed by one reviewer (LE) using the Cochrane recommended Quality in Prognosis Studies (QUIPS) [12]. This tool assesses the following domains: 1) study participation; 2) study attrition; 3) measurement of predictive factors; 4) adjustment for confounding; 5) measurement of outcomes; and 6) appropriateness of statistical analyses. Similar to previous systematic reviews regarding predicting factors for AIS [13,14], we applied the modified QUIPS by excluding domain 4 because our goal was to estimate the probability of the outcome rather than explore causality between factors and the outcome (Table 1a). Thirteen items were point-scored with either 1, if satisfactorily described or 0 in case of missing data or insufficient information. A score of ≥9 was considered high-quality and <9 low-quality.

**Table 1:** Quality assessment based on the QUIPS tool.

a. Criteria														
Quality criteria														Max score
**Study participations**														
A) Description of source population														1
B) Description of inclusion and exclusion criteria														1
C) Sufficient description of baseline characteristics (age, gender, severity of pectus and scoliosis and whether syndromic disorders were included)														1
**Study attrition**														
D) Follow-up until maturity														1
E) Prospective or retrospective data collection														1
F) Loss to follow-up ≤ 20%														1
G) Information about loss to follow-up														1
**Measurement of prognostic factors**														
H) Exposure assessment blinded for the outcome														1
I) Exposure measured identically in the studied population at baseline and follow-up														1
**Measurement of outcome**														
J) Outcome assessment blinded for exposure														1
K) Outcome measured identically in the studied population at baseline and follow-up														1
**Analysis approach**														
L) Measure of association (p-value) or other measures of variance given.														1
M) Analysis adjusted for confounding factors														1

b. Results														
Author, year	Total score	A	B	C	D	E	F	G	H	I	J	K	L	M
Waters, 1989	6	1	0	1	0	1	1	0	0	0	0	1	0	1
Nagasao, 2010	6	1	0	0	0	1	1	0	0	1	0	1	1	0
Hong, 2011	5	0	0	1	0	0	0	0	0	1	0	1	1	1
Wang, 2012	6	0	1	0	0	1	0	0	0	1	0	1	1	1
Chung, 2016	9	1	1	1	0	1	1	0	0	1	0	1	1	1
Choi, 2016	7	1	1	0	0	1	1	0	0	1	0	0	1	1
Ghionzoli, 2016	7	1	0	1	0	1	0	0	0	1	1	1	1	0
Park, 2017	10	1	1	1	0	1	1	0	0	1	1	1	1	1
Tomaszewski, 2017	6	0	0	1	0	1	1	0	0	1	0	1	1	0
Zhong, 2017	6	1	1	0	0	1	0	0	0	1	0	1	1	0
Tauchi, 2018	9	1	1	1	0	1	0	1	1	1	0	0	1	1
Iscan, 2020	7	1	0	0	0	1	1	0	0	1	1	1	1	0
Alaca, 2021	9	1	1	1	0	1	1	1	0	1	0	1	1	0

*studies scoring ≥9 points are considered of high quality.

#### Category II

No methodological appraisal was performed because most prevalence concerned a population characteristic or a secondary outcome. We believed it inaccurate to use the methodological quality of the main study for secondary values. Instead, a reported prevalence was considered probable only if diagnostic measures were described and the study population comprised >50 participants, and zero syndromic cases.

#### Category III

These studies were narratively reviewed.

### Statistical analysis

#### Category I

Provided that the studies matched sufficiently regarding study design, population, outcome, and statistical analysis, correlation coefficients were statistically pooled. In case of excessive heterogeneity among studies, the level of evidence of the reported predictive factors was assessed according to a best evidence synthesis (BES) [15]. Evidence was considered *strong* if findings were consistent (>75%) in multiple (≥2) high-quality studies, *moderate* if the finding was present in 1 high-quality study and consistent (>75%) in multiple (≥2) low-quality studies, *limited* if the finding was present in 1 high-quality study or consistent (>75%) in ≥3 low-quality studies, *inconclusive* if findings were found in <3 low-quality studies, and *conflicting* if <75% of the studies reported consistent findings [14]. Predictive factors for the prevalence of AIS in PD patients and the influence of PD correction on the concomitant prevalence of scoliosis as well as on the postoperative CA are reported separately.

#### Category II

Any reported prevalence (category I and II combined) was collected. Ranges and mean values were manually calculated for the pooled data and per group (PE, PC, age of 0-32 years, idiopathic population, large sample size, and probable prevalence).

## Results

### Search

**Figure 1:**
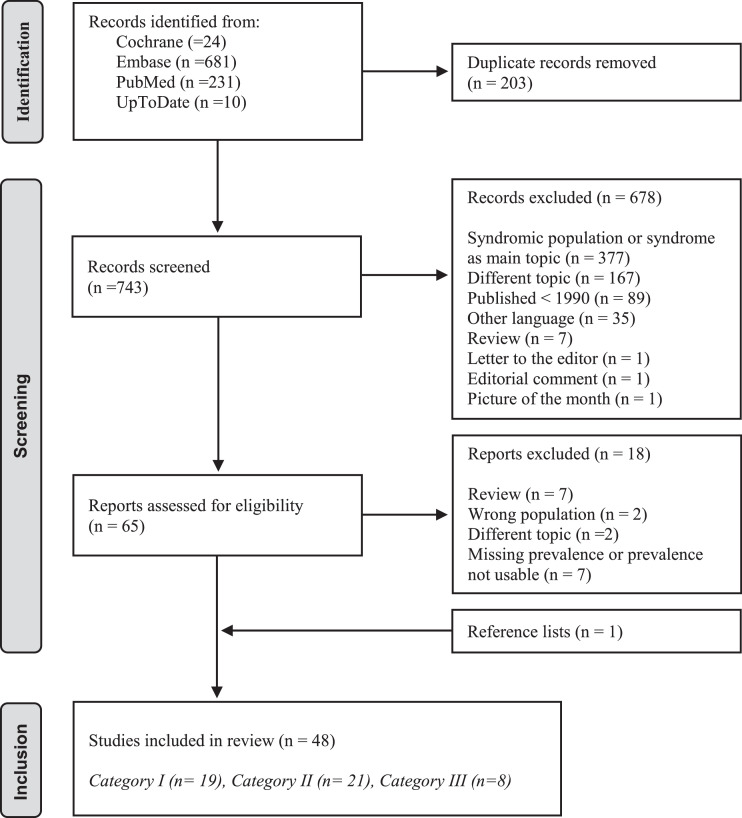
PRISMA 2020 flow diagram of identification, screening, and inclusion of papers in the 3 categories.

The search yielded 946 studies. Of 743 unique studies, 65 remained eligible and were fully read. One study was identified by backward citation. Eventually, 48 studies were included: 19 in category I [5,9,[16], [17], [18], [19], [20], [21], [22], [23], [24], [25], [26], [27], [28], [29], [30], [31], [32]], 21 in category II [33], [34], [35], [36], [37], [38], [39], [40], [41], [42], [43], [44], [45], [46], [47], [48], [49], [50], [51], [52], [53], and 8 in category III [54], [55], [56], [57], [58], [59], [60], [61] (Figure 1).

### Study characteristics (appendices B and C)

#### Category I

Nineteen studies focused on the association between PD and scoliosis, of which 4 described cases [9,20,24,27], 5 described spinal change after PD correction [19,21,23,25,26], and 2 PD change after scoliosis correction [17,28]. Thirteen studies analyzed predictive factors [5,16,18,19,[21], [22], [23],^25^,^26^,[29], [30], [31], [32]], of which three solely included patients with PD-associated scoliosis [25,29,32] (Appendix B). Five of these 13 studies did not describe inclusion criteria regarding either scoliosis or syndromic cases [16,18,23,25,32]. Six definitions for PE severity were used. The populations were predominated by PE and male gender (3:1). Population sizes ranged from 25 to 468 patients. The number of patients with PD concomitant scoliosis ranged from 6 to 99. Considering these heterogeneities, pooling was not possible, and evidence was combined according to a BES.

#### Category II

Combining categories I and II, 31 studies reported a prevalence of scoliosis in PD populations (Appendix C). In all, 19 comprised solely PE cases [18,19,[21], [22], [23],^26^,^30^,^31^,^34^,^36^,[38], [39], [40], [41], [42],^45^,^47^,^49^,^50^], 2 solely PC cases [35,44], and 6 analyzed PE and PC separately [5,16,35,43,46,51,52]. Fourteen studies described definitions of scoliosis or its diagnostic measures [5,18,19,22,23,26,30,31,33,34,40,47,52,53], 9 involved an entire idiopathic population [16,19,22,26,30,31,34,41,51,52], and 1 a non-surgical one [53].

### Quality assessment

Of the 13 studies assessed, 4 studies considered high-quality [16,19,26,29]. Nearly all methodological shortcomings concerned the lack of follow-up (item D), information about the loss to follow-up (items F and G), and blinding (items H and J) (Table 1b).

### Study results and BES

#### Category I

##### Association between PD and AIS

All studies reported a significantly higher prevalence of scoliosis in PD patients as compared to the general population. Most PD-associated AIS involved single curves of the middle to lower thoracic spine (Lenke type 1) [5,22,[29], [30], [31]]. Regarding convexity (right- versus left-sided scoliotic curve), the literature was contradictory [21,31]. One study reported a significant association between PE severity and the rotation degree of thoracic vertebrae [30].

Three studies proposed novel classifications for PD and compared these different types to the prevalence and severity of AIS [18,29,32]. Choi et al. retrospectively categorized 230 PE patients by clustering 5 statistical variables. Of the 7 newly identified PE subgroups, scoliosis was more frequently associated with the subgroup representing a double distorted sternum (p = 0.008) [18].

##### BES of the predictive factors for the development of AIS in PD patients (Table 3)

Fifteen factors were analyzed (Appendix D). Moderate evidence suggested that higher age was associated with a higher prevalence of AIS, as most patients undergoing correction of PD with concomitant scoliosis were older [18,22,26,31,32]. Moderate evidence also suggested that PD asymmetry was not associated with prevalence of AIS. Furthermore, sternal tilt angle was not associated with increased CA. Concerning the association between HI and AIS, the evidence was conflicting.

##### Influence of PD correction on scoliosis

In the literature, both improvement and aggravation of scoliosis were reported following PE correction. No therapeutic studies involved PC.

Particularly the development or progression of scoliosis after PE correction was described in case reports and series. For the described cases of scoliosis de novo, brace therapy and Nuss bar removal achieved satisfactory results [9,24,27]. The cases that showed progression warranted scoliosis surgery 3 months after the Nuss procedure [20]. Additionally, 2 cohort studies reported a postoperative increase of CA in 1.1% to 4.5% of cases [21,42].

The cohort of Ghionzoli et al., comprising 34 PD patients with mild to moderate AIS showed an overall decrease of 1.5°CA 3 years after the Nuss procedure [21]. The largest retrospective study (n = 63) consisting of PD-associated scoliosis patients only was by Chung et al. In their cohort, 41/63(65%) experienced a decrease of CA after PE surgery, while 22/63(35%) experienced a progression of CA [19]. In the study of Park et al., who followed 468 thoracic curvatures (<10°CA included) pre- and post-PE correction, the prevalence of AIS was 44/468(9.4%) preoperatively and 46/468(10%) postoperatively. Though, they also reported that 22/44(50%) patients with a pre-corrective scoliosis did not have a CA of >10° after correction of their PE, and 24/46(52%) patients with post-corrective scoliosis developed a CA of >10° after correction of PE [26].

Finally, Nagasoa et al. evaluated thoracic transformation after the Nuss procedure by comparing 4 groups based on right/left convexity of the thorax and right/left bowing of the spine in 25 patients with asymmetric PE. Spinal curvature postoperatively straightened significantly when, preoperatively, the side of spinal bowing coincided with the side of anterior wall concavity (right-right versus right-left, p = 0.002, and left-right versus left-left, p = 0.033) [25]. Their theory was not confirmed when repeated in the cohort (63 patients with PD-associated AIS) of Chung et al.[19].

##### BES of predictive factors for the prevalence of AIS and difference in CA after correction of PD

Twelve factors involving postoperative curvature change in PD patients were analyzed (Appendix E). Concerning the prevalence of AIS after PD correction, only female gender was a predictive factor, by a limited amount of evidence (Table 4). Strong evidence suggests that an older age at PD correction is not associated with an increase of CA postoperatively (Table 5). Furthermore, preoperative CA was associated with neither a reduction nor an increase in CA postoperatively. There was limited evidence for gender and preoperative HI being predictive factors for postoperative increase of CA.

##### Influence of scoliosis correction on PE

One cohort study examined 20 PE patients undergoing scoliosis correction, of which 8 had AIS. In 1 of the AIS patients, the HI deteriorated, while overall the postoperative HI improved by 0.46° [28]. Additionally, a case report described another case of HI deterioration after scoliosis correction. This case also acquired respiratory symptoms postoperatively, which resolved after an additional Nuss procedure [17].

#### Category II

##### Prevalence

Thirty-one studies, comprising 12 052 PD patients, reported a prevalence of mixed idiopathic/syndromic scoliosis ranging from 4%-52% (mean 28.6%). Classification of the studies on characteristics showed a lower mean prevalence for pure PE, PC, and in the AIS population (25.4%, 21.9%, and 22.8%, respectively). The 7 studies reporting a probable prevalence comprised 1835 PE patients and showed a mean prevalence of scoliosis of 13.1% (range 8.1%-50.7%) (Table 2 and Appendix C).

**Table 2:** Analysis of the prevalence of scoliosis in pectus deformities.

Group	No. Of studies	No. Of patients	Mean (%)	Range (%)
*All*	31	12052	20.1	4.0-52.0
*PE*	25	6725	19.9	3.5-50.7
*PC*	8	685	23.5	4.4-60.0
*Age 0-32 years*	23	5421	19.4	4.6-52.0
*Idiopathic population*	11	2146	21.0	5.0-52.0
*n > 300*	9	9785	23.1	8.1-43.3
*Probable prevalence^a,b^*	7	1835	19.6	8.1-50.7

a: defined as n ≥ 50, exclusion of syndromic disorders and described diagnostic method.

b: comprising only PE by coincidence

**Table 3:** Level of evidence for the predictive factors for AIS in PD patients.

**Strong evidence**^a^			
No predictive factors presented strong evidence			
**Moderate evidence**^b^			
Prevalence of AIS		Higher CA	
Associated	Not associated	Associated	Not associated
• Age at pectus correction	• Pectus asymmetry		• Sternal Tilt angle
**Limited evidence**^c^			
Prevalence of AIS		Higher CA	
Associated	Not associated	Associated	Not associated
• Number of inserted bars during Nuss • Pectus severity*	• Body Mass Index • Gender	• Number of inserted bars during Nuss • Height • Weight	• Body Mass Index • Pectus type
**Inconclusive evidence**^d^			
Prevalence of AIS		Higher CA	
Associated	Not associated	Associated	Not associated
	• Age at diagnosis • Angle of Louis • Flatness Index • Family history		
**Conflicting evidence**^e^			
• Pectus type • Haller Index • Sternal Tilt angle		• Age at pectus correction • Gender • Haller index • Pectus asymmetry	

*measured using a caliper

a, Consistent (>75%) findings in multiple (≥2) high-quality studies

b, Findings in one high-quality study and consistent (>75%) findings in multiple (≥2) low quality studies

c, Findings in one high-quality study or consistent (>75%) findings in ≥3 low-quality studies

d, Findings found in <3 low-quality studies

e, <75% of the studies reported consistent findings

**Table 4:** Level of evidence for the predictive factors for a higher prevalence of AIS after PD correction.

**Strong evidence**^a^	
No predictive factors presented strong evidence	
**Moderate evidence**^b^	
No predictive factors presented moderate evidence	
**Limited evidence**^c^	
Associated	Not associated
• Female gender	
**Inconclusive evidence**^d^	
Associated	Not associated
	• Preoperative CA • Preoperative HI • Preoperative STA • Preoperative Asymmetry Index
**Conflicting evidence**^e^	
• Age at pectus correction	

a, Consistent (>75%) findings in multiple (≥2) high-quality studies

b, Findings in one high-quality study and consistent (>75%) findings in multiple (≥2) low quality studies

c, Findings in one high-quality study or consistent (>75%) findings in ≥3 low-quality studies

d, Findings found in <3 low-quality studies

e, <75% of the studies reported consistent findings

**Table 5:** Level of evidence for the predictive factors of transformation of spinal curvature after PD correction.

**Strong evidence**^a^			
Decrease of CA		Increase of CA	
Associated	Not associated	Associated	Not associated

	• Preoperative CA		• Age at pectus correction
**Moderate evidence**^b^			
No predictive factors presented moderate evidence			
**Limited evidence**^c^			
Decrease of CA		Increase of CA	
Associated	Not associated	Associated	Not associated
	• Height • Weight • Body Mass Index • AIS convexity • Preoperative HI • Preoperative STA • Period of bar implementation	• Height • Weight	• Gender • AIS convexity • Preoperative CA • Preoperative HI • Preoperative STA • Number of inserted bars during Nuss • Period of bar implementation
**Inconclusive evidence**^d^			
Decrease of CA		Increase of CA	
Associated	Not associated	Associated	Not associated
	• Preoperative Asymmetry Index		• Preoperative Asymmetry Index
**Conflicting evidence**^e^			
• Age at pectus correction • Gender • Number of inserted bars during Nuss			

a, Consistent (>75%) findings in multiple (≥2) high-quality studies

b, Findings in one high-quality study and consistent (>75%) findings in multiple (≥2) low quality studies

c, Findings in one high-quality study or consistent (>75%) findings in ≥3 low-quality studies

d, Findings found in <3 low-quality studies

e, <75% of the studies reported consistent findings

#### Category III

##### Genetics

Familial inheritance was discussed in 5 studies [5,46,[54], [55], [56]]. A positive family history for AIS was reported in 5%-20% of PE patients and in 5%-12.5% of PC patients [5,46,55,56]. PD-associated AIS was reported in respectively 0.9%-9% and 6.1% [5,56]. Creswick et al. found strong evidence for a common association of PE and AIS in the 4-generation pedigrees of 34 families (1433 individuals) [54].

Gurnett's DNA analysis revealed a novel locus for AIS and PE on chromosome 18q12.1-q12.2 [56] and Karner found involvement of chromosome 6q24.4. Their induced deficiency of the G protein-coupled receptor (Gpr)126 (encoded by the ADGRG6 gene and previously linked to AIS) resulted in 1) a loss of cartilage quality, manifesting as AIS and PE and 2) the up-regulation of Gal3st4, a protein likewise linked to PE [57].

##### Etiology

Five studies focused on the change of thoracic pressure distribution after PD correction [25,58,[60], [61], [62]]. CT models correctly predicted surgical outcome [25,61,62]. With these digital and corresponding 3D printed models, the authors demonstrated that simultaneous surgical correction of AIS and PE could be successful, but that this also influenced the deformation and stress of the thorax, affecting breathing and heartbeat [60].

## Discussion

The purpose of this review was to evaluate the clinical relevance of concomitant PD and AIS by examining the prevalence of AIS in association with PD, and to assess factors associated with the presence and severity of AIS in this population.

In our opinion, the last review (published 32 years ago) needed updating because Waters et al. included non-idiopathic cases and defined scoliosis as being a lateral curvature of >5° [5], which differs from the currently upheld definition of the Scoliosis Research Society as being a lateral curvature of ≥10° CA. Based on the present definition of scoliosis, only 11.4% of their PE and 6.2% of PC patients would have been diagnosed with AIS. Not the 21.5% that is cited throughout literature.

### Association

Our search yielded 49 studies that verified an association between PD and AIS. There is no consensus on the correlation between the severity of PD and AIS. Studies have found evidence to support [16,22,29,30,32], and oppose [18,26,31,32] the theory. Concerning age and location of spinal deformation, idiopathic PD-associated AIS seems to mimic normal AIS.

Age exhibited the second-highest level of evidence as a predictive factor, meaning PD-associated AIS also progresses with age [63], and the deformation most frequently involved the lower half of the thoracic spine, as in 88% of AIS cases [64].

We found no association between a higher prevalence of PD-associated scoliosis and female gender. However, predominance of PD in the male population [6,30] could be a confounder when investigating gender as a predictive factor for AIS.

Independent studies showed that indices such as sternal displacement to the left or double distortion (downward bending of the proximal part and counter-clockwise rotation of the distal part) seem interesting predictive factors for further research [18,29].

### Influence of operative treatment

The literature provided evidence that the severity of concomitant scoliosis may be influenced by PD correction. Studies have shown an increase of CA [20,21,23,[25], [26], [27], [28],^42^], a decrease of CA [19,21,25,26], a complete disappearance of scoliosis [26], as well the development of scoliosis [9,24,26].

Considering this review has established that PD-associated scoliosis progresses with age, a postoperative development of scoliosis is not necessarily due to the Nuss procedure. However, the reported CA increase cannot be fully explained by the natural course of AIS. Floccari et al. reported an aggravation rate in fully grown adolescents and twice as high as in progressive AIS's most severe form [20,63]. Moreover, it was demonstrated that the metallic bars used in the Nuss procedure administer asymmetric pressure on the thorax [25,58,60,61]. In addition, cases were reported in which a postural preference, because of postoperative pain, provoked AIS [27] and in which Nuss-acquired scoliosis improved immediately after bar removal [24].

Age did not appear to be a predictive factor for a higher prevalence of postoperative scoliosis. Moreover, age at PD correction is not at all associated with an increase in postoperative CA, based on 3 studies that measured age on a continuous scale [19,23,26]. When divided into age groups, the study by Park et al., which comprised 44 idiopathic PD-associated AIS patients, showed that if patients underwent correction above 10 years of age, they experienced a significant increase of CA postoperatively [26]. Yet, Iscan et al. found opposite results when evaluating 100 spines after a Nuss procedure – especially in children: Their mean CA progressed from 3.7° to 4.7° after PE correction. However, because the preoperative CA of most patients was <10°, the ultimate change in CA had no clinical significance [23].

Two other frequently described predictive factors for postoperative aggravation of scoliosis were severity of preoperative PE (HI of ≥3.5) and scoliosis (CA of >15°) [19,23]. Chung et al., who compared factors between patients who suffered from an increase of CA to those in whom CA decreased, showed that scoliosis with a preoperative CA of <15° improved after sternum correction, while scoliosis with a preoperative CA of >15° aggravated postoperatively (3.86° vs. −2.88°, p < 0.001) [19]. Park et al.’s multivariate analysis likewise showed a significant correlation between a high preoperative CA and an increase of CA after PD correction [26]. Our BES could neither confirm nor reject their hypothesis, but the mentioned thresholds for HI and CA might suggest that cut-off values may exist beyond which correction of PD will aggravate scoliosis. Establishing such thresholds is a focus for further research.

*Vice versa*, scoliosis correction could improve or worsen the severity of PD [28]. However, no data are available on this issue, as most authors recommend performing PE correction first to avoid possible perioperative hemodynamic instability [65], [66], [67].

Unfortunately, no PD studies have focused on the treatment of concomitant scoliosis.

### Prevalence

Due to the heterogeneity of the studies, it is difficult to derive a valid and reliable percentage that represents the true prevalence. Our presumed prevalence of scoliosis in PE patients ranges from 8.1% to 50.7%, with a mean of 13.1% [19,22,26,30,31,47].

This value may be underestimated due to limited follow-up data after PD correction or because of the PD correction itself. Though, this implies that PD develops earlier in life than AIS does.

This value might be overestimated, as nearly all study populations comprised patients screened for surgery at tertiary referral hospitals leaving out patients that were not or conservatively treated for PD. Moreover, in healthy school children, PD-associated scoliosis was only present in 5%-10% of cases [53,68].

Altogether, the prevalence of AIS in children without PD is lower [1,16].

### Etiology

It remains unclear why scoliosis occurs more often when an anterior chest wall deformity exists. Though genetic components are strongly suspected and up to 10% of the family members of PD patients with concomitant AIS have either PD or AIS [5,46,54,56,57], most hypotheses appeal to the mechanical forces of the thorax. Such as the imbalance caused by asymmetric pressures of the intrathoracic organs, the paraspinal muscles, and growth itself [5,18,26,31]. One of these explanations is the anatomical position of the heart [5,22,31,32]. The deformed sternum pushes the heart to the left, and the heart pushes the thoracic vertebrae to the right. Two studies confirm this hypothesis by finding that scoliosis was mostly located at the same axial level as PE [32] and that PD severity was a significant risk factor for more severe vertebral rotation [30].

### Limitations

The major limitation of this review is the heterogeneity of the studies and parameters representing one predictive factor. It was therefore impossible to perform a meta-analysis. When interpreting our results, one must keep in mind that some populations of the category I and II studies did include syndromic cases (Appendix B and C).

Furthermore, the methodological quality of most studies was low. Almost all were retrospective, not blinded, and lacked control groups, resulting in *inconclusive* or *conflicting* evidence for most predictive factors.

An important limitation of a BES is that although a singular study of high quality can provide the third-highest level of evidence, when combined with an opposing low-quality study, the level of evidence is reduced to *conflicting*.

Finally, most discovered prevalence may be biased because nearly all were reported as a secondary outcome, and their definitions were rarely specified.

Nonetheless, at present, this is the only review combining all existing knowledge on PD-associated AIS. Future studies should include non-surgical populations, repetition of previous research, inclusion of control groups, and differentiation between PE and PC.

## Conclusions

Current literature confirmed the association between PD and AIS in patients with an indication for PD correction. PD correction surgery can affect the severity of concomitant scoliosis, and *vice versa*. Our results indicate that severe concomitant scoliosis, opposite asymmetry, and older age may require extra attention when planning PD correction in a patient with concomitant AIS. 3D models to predict surgical outcomes have been developed but need more research to be implemented in daily clinical practice.

## Declaration of Competing Interest

The authors declare that they have no known competing financial interests or personal relationships that could have appeared to influence the work reported in this paper.

## Appendix Supplementary materials

Image, application 1

Image, application 2

Image, application 3

Image, application 4

Image, application 5
