# Supplementary material for: Clinical significance of concomitant pectus deformity and adolescent idiopathic scoliosis: systematic review with best evidence synthesis
Source: N Am Spine Soc J. 2022 Jun 25;11:100140. doi: 10.1016/j.xnsj.2022.100140 (PMC9256832; doi:10.1016/j.xnsj.2022.100140)
Supplement: Supplementary file 1 [file mmc1.docx]

Appendix A. Searches and hits per database on the 12th of December 2021.

| **Database** |  | **Search** | **Hits** |
| --- | --- | --- | --- |
| Cochrane |  | (MeSH descriptor [scoliosis] explode all trees OR ‘’scoliosis’’) **AND** (MeSH descriptor [funnel chest] explode all trees OR MeSH descriptor [pectus carinatum] explode all trees OR ‘’pectus excavatum” OR “funnel chest” OR ‘’funnel thorax’’ OR ‘’funnel breast’’ OR ‘’pectus carinatum” OR “pigeon breast” OR ‘’chicken breast’’) | 24 |
| Embase |  | scoliosis/exp or scoliosis:ti,ab,kw **AND** ('funnel chest'/exp OR (Funnel:ti,ab,kw AND (chest or breast or thorax):ti,ab,kw (pectus and excavatum):ti,ab,kw) **OR**  ('pigeon thorax'/exp OR ((pigeon or chicken) AND (thorax or breast or chest)) OR pectus:ti,ab,kw and (carinatum or carinatus):ti,ab,kw) | 681 |
| PubMed |  | ("Scoliosis"[Mesh] OR "Scoliosis"[tiab]OR "Scolioses"[tiab] OR AIS[tiab] OR AIS[ot]) **AND** ("Funnel Chest"[Mesh] OR pectus[ot] OR (Funnel[tiab] AND (chest*[tiab] OR breast*[tiab] OR thorax[tiab])) OR (Pectus[tiab] AND (Excavatum[tiab] OR excavatus[tiab] OR deform*[tiab])) **OR** "Pectus Carinatum"[Mesh] OR (Pectus[tiab] AND (Carinatum[tiab] OR carinatus[tiab])) OR ((pigeon[tiab] OR chicken[tiab]) AND (breast*[tiab] OR chest*[tiab] OR thorax[tiab]))) | 231 |
| UpToDate |  | 1. Pectus excavatum: Etiology and evaluation- physical examination - Thoracic abnormalities | 5 |
|  |  | 2. Pectus carinatum: clinical features - associated abnormalities | 4 |
|  |  | 3. Adolescent idiopathic scoliosis: Clinical features, evaluation, and diagnosis | 1 |
|  |  | 4. Scoliosis in the adult | 0 |
| **Total** | | | **946** |
