# Supplementary material for: Clinical significance of concomitant pectus deformity and adolescent idiopathic scoliosis: systematic review with best evidence synthesis
Source: N Am Spine Soc J. 2022 Jun 25;11:100140. doi: 10.1016/j.xnsj.2022.100140 (PMC9256832; doi:10.1016/j.xnsj.2022.100140)
Supplement: Supplementary file 2 [file mmc2.docx]

Appendix B. Characteristics of studies researching the association between PD and AIS (category I).

| **Author, year** |  |  | **Population** | | | | **Definitions and diagnostic measure** | | **Treatment for scoliosis** | **Conclusion about association** |
| --- | --- | --- | --- | --- | --- | --- | --- | --- | --- | --- |
|  | Level of evidence | Deformity | Size Total | Both PD and scoliosis | Idiopathic cases | Age (years) | Pectus deformity | Scoliosis |  |  |
|  |  | *(PE/PC)* | *N=* |  | *% of total* | *Mean, SD (range)* |  |  |  | *(yes/no)* |
| Waters, 1989 | IV | PE | 461 | 99 | 93% | 9.6 (2-31) | Welch index (X-ray) | lateral curvature >5° (AFBT and X-ray) | 18% brace and 7% add. Surgery | yes |
|  |  | PC | 131 | 28 | 82% | 13 (3-24) | na (X-ray) | lateral curvature >5° (AFBT and X-ray) | 11% bracing and 3.6% surgery. |  |
| Nagasoa, 2010 | III | PE | 25 | 25 | unsure | 11.6 | HI (CT) | CA >10° (CT) | nm (CA >30° was exclusion criteria) | nm |
| Hong, 2011 | III | PE | 248 | 56 | 100% | 16.4 ± 7.1 | HI >3.5 (CT) | coronal curvature >10° (CT) | nm | yes |
| Wang, 2012 | III | PE | 142 | 25 | 100% | 14 ± 5.4 (3-32) | HI >3.2 (CT and X-ray) | CA >10° (CT and X-ray) | nm | yes |
| Chung, 2016 | III | PE | 63 | 63 | 100% | 16.9 | HI >3.2 (CT and X-ray) | CA >10° (CT and X-ray) | nm | yes |
| Choi, 2016 | III | PE | 230 | 17 | 97% | 6.0 (2.1-30.1) | HI (CT) | nm (CT) | nm | yes |
| Ghionzoli, 2016 | II | PE | 67 | 34 | 100% | 15.6 (12-21) | HI (MRI) | CA >10° (X-ray) | all conservative | yes |
| Park, 2017 | III | PE | 468 | 44 | 100% | 6.8 ± 4.9 (3-20) | HI (CT and X-ray) | CA >10° (CT and X-ray) | no (exclusion criteria) | yes |
| Tomaszewski, 2017 | IV | PE | 54 | 8 | 100% | 13.6 ± 3.0 | HI (CT) | CA >10° (CT and X-ray) | nm | yes |
| Zhong, 2017 | III | PE | 37 | 28 | unsure | (4-44) | HI > 3.25 (CT) | CA >10° (CT and X-ray) | nm | yes |
| Tauchi, 2018 | III | PE | 70 | 70 | 59% | 10.3 (1-19) | Modified HI >3.25 (CT) | CA >10° (CT) | 27 surgery and 14 cast/brace | nm |
| Iscan, 2020 | III | PE | 100 | 6 | unsure | 19.6 ± 6.7 | HI >3.25 (CT) | CA >10° (X-ray) | physiotherapy recommendation | yes |
| Alaca, 2021 | II | PE and PC | 180 | 9 | 100% | 14.2 | By Caliper as of Ewert^1^ (MRI) | nm (clinical and MRI) | nm | yes |
| AFBT = Adams Forward Best Test, CT = computed tomography scan, HI=Haller Index, MRI = magnetic resonance imaging, na=not applicable, nm = not mentioned, PC = pectus carinatum, PE = pectus excavatum.  * calculated by Welch score.  **^1.^** Ewert F, et al. Does an external chest wall measurement correlate with a CT-based measurement in patients with chest wall deformities? *Journal of Pediatric Surgery.* 2017;52:1583-1590. | | | | | | | | | | |
