# Supplementary material for: Clinical significance of concomitant pectus deformity and adolescent idiopathic scoliosis: systematic review with best evidence synthesis
Source: N Am Spine Soc J. 2022 Jun 25;11:100140. doi: 10.1016/j.xnsj.2022.100140 (PMC9256832; doi:10.1016/j.xnsj.2022.100140)
Supplement: Supplementary file 3 [file mmc3.docx]

| Appendix C. Characteristics of studies comprising pectus patients stating a prevalence of scoliosis. | | | | | | | | |
| --- | --- | --- | --- | --- | --- | --- | --- | --- |
| **Author** | **No. of patients** | **Population** Deformity | Age (mean, range) | Syndromes included | **Definitions** Pectus | Pectus | **Prevalence of Scoliosis** |  |
| Waters, 1989 | 461 | PE | 9,6 (2-31) | 13% | Welch index | lateral curvature >5° | **21.5%** |  |
| Akçali, 1999 | 43 | PE 24 \| PC 13 \| mixed 6 | 14,4 (5-23) | 29.7% | Welch index | lateral curvature >5° | **13.5%** |  |
| Mandhan, 1999 | 129 | PE 114 \| 5 PC | 0 to 15 | ~31.5% | ­nm | ­nm | **23.0%** |  |
| Schoenmakers, 2000 | 21 | PE 16 \| PC 5 | 12.2 (5.7-16.7) | no | ­nm | positive AFBT | **52.0%** |  |
| Coln, 2002 | 8 | PE | 24.0 (19-46) | no | HI > 3.25 | ­nm | **14.0%** |  |
| Koumbourlis, 2004 | 103 | PE | 5 to 19 | no | ­nm | ­nm | **22.0%** |  |
| Kelly, 2007 | 327 | PE | 0 to 21 | 7.3% | HI >3.2 | ­nm | **21.0%** |  |
| Fonkalsrud, 2008 | 260 | PC | 18.2 (3-65) | 8% | ­nm | ­nm | **32.0%** |  |
| Fonkalsrud, 2009 | 912 | PE | 19.8 (2.5-67) | ~10% | ­nm | ­nm | **39.0%** |  |
| Westphal, 2009 | 26 | PE 17 \| PC 9 | 12.03 (11-14) | unsure | of Herbert^1^ | nm | **11.5%** |  |
| Haje, 2009 | 4012 | PE 901 \| PC 3111 | children and adults | 0.3% | of Herbert^1^ | ­nm | **43.3%** |  |
| Luu, 2009 | 48 | PE | 28 (16-54) | ­8.3% | HI >2.5 | ­nm | **4.0%** |  |
| Prats, 2009 | 18 | PE | 18 | 37% | HI > 3.25 | ­nm | **38.9%** |  |
| Kelly, Robert, 2010 | 1215 | PE | 12.0 (1-31) | 38.6% | clinical, HI > 3.2 | ­nm | **28.0%** |  |
| Hong, 2011 | 248 | PE | 11.1 (4.3-18.5) | no | HI >3.5 | CA >10° | **22.6%** |  |
| Wang, 2012 | 142 | PE | 14.0 (3-32) | no | HI >3.2 | CA >10° | **17.6%** |  |
| Lopez, 2013 | 61 | PC | 13.5 (5–25) | 1.6% | na | nm | **14.0%** |  |
| Kuru, 2015 | 88 | PE | 18.4 (14-29) | 41% | HI >2.2 | ­nm | **17.0%** |  |
| McHugh, 2016 | 77 | PE | 16.5 (6.2-43) | ­unsure | HI | CA >10° | **14.3%** |  |
| Kuru, Cakiroglu, 2016 | 280 | PE 207 \| PC 73 | 19.7 (8-47) | 25,1% | HI | ­nm | **9.0%** |  |
| Chung, 2016 | 779 | PE | 16,9 | no | HI >3.2 | CA >10° | **8.1%** |  |
| Choi, 2016 | 230 | PE | 6.0 (2.1-30.1) | 3% | HI | ­nm | **7.4%** |  |
| Ghionzoli, 2016 | 67 | PE | 16.5 (12–26) | no | HI | CA >10° | **50.7%** |  |
| Tomaszewski, 2017 | 54 | PE | 13,6 | no | HI | CA >10° | **14.8%** |  |
| Park, 2017 | 468 | PE | 6.8 (3-20) | no | HI | CA >10° | **9.4%** |  |
| Murphy, 2018 | 442 | PE 409 \| PC 24 \| mixed 9 | 15.5 | 10% | HI | ­nm | **8.4%** |  |
| Kelly, 2020 | 1034 | PE | 6 to 46 | 11.8% | Clinical, HI > 3.2 and CI >10% | ­nm | **29.0%** |  |
| Işcan, 2020 | 100 | PE | 19.6 | unsure | HI >3.2 | CA >10° | **6.0%** |  |
| Okuyama, 2021 | 8 | PE | 16.2 (8-20) | unsure | HI | nm | **25%** |  |
| Alaca, 2021 | 180 | 90 PE \| 90 PC | 14.5 (10-18) | no | HI and by caliper | nm | **5%** |  |
| Ramadan, 2021 | 76 | 30 PE \| 46 PC | 14.2 | no | HI | nm | **14.5%** |  |
| *AFBT = Adams Forward Best Test, CA = Cobb's Angle, CI= correction index, CT= computed tomography scan, HI= Haller Index, MR = medical records, MRI = Magnetic Resonance imaging, na = not applicable, nm = not mentioned (missing data), PE= pectus excavatum, PC= pectus carinatum. ­* | | | | | | | | |

*^1^ Herbert SK et al., Orthopedia e Traumatologia-: Principios e Prática: Artmed Editora; 2016.*
