# Supplementary material for: Clinical significance of concomitant pectus deformity and adolescent idiopathic scoliosis: systematic review with best evidence synthesis
Source: N Am Spine Soc J. 2022 Jun 25;11:100140. doi: 10.1016/j.xnsj.2022.100140 (PMC9256832; doi:10.1016/j.xnsj.2022.100140)
Supplement: Supplementary file 4 [file mmc4.docx]

Appendix D. Determinants studied as predictors for pectus-associated scoliosis.

| Reported determinant | References | Study quality | Measurement method | Statistical analysis (prevalence \| severity) | Association with scoliosis | Association with higher CA. |
| --- | --- | --- | --- | --- | --- | --- |
| Age at diagnosis (in years) | Waters | Low | Continuous | np | o | x |
| Age at pectus correction | Hong | Low | Continuous | P <0.0001 \| P = 0.612 | + | o |
| (in years) | Wang | Low | Groups (≤10y, 11~17, ≥18) | P <0.05^2^ \| np | + | x |
|  | Choi | Low | Continuous | P = 0.020 \| np | + | x |
|  | Park | High | Continuous | P < 0.001 \| P < 0.001 | + | + |
|  | Zhong | Low | Groups (< 18y versus ≥ 18y) | P = 0.017 \| P = 0.044 | + | + |
| Gender | Hong | Low | Dichotomous | P = 0.002 \| p = 0.227 ^6^ | + | o |
| (female vs male) | Tomaszewski | Low | Dichotomous | np^1^ | - | + |
|  | Choi | Low | Dichotomous *(Male risk)* | P = 0.389 \| np | o | x |
|  | Park | High | Dichotomous | P = 0.186 \| P*=* 0.288 | o | o |
| Pectus type | Choi | Low | 7-type CT assisted classification | P = 0.008 \| np | + | x |
|  | Zhong | Low | Vertical distribution (groups Th1-6 versus Th7-12) | P = 1.0 \| P > 0,05 | o | o |
|  | Tauchi | High | Right/center/left sided | np | o | o |
| Height | Park | High | Continuous | np \| P=0.002 | x | + |
| Weight | Park | High | Continuous | np \| P=0.009 | x | + |
| BMI | Park | High | Continuous | P = 0.010 \| P = 0.719 | - | o |
| Haller Index | Hong | Low | Continuous | P = 0.061 \| P = 0.895 | o | o |
|  | Wang | Low | Groups (<3.2, ≤3.2 - < 3.5, ≥ 3.5) | P < 0.05^3^ \| np | + | x |
|  | Chung | High | Not reported | np \| P = 0.011 | x | + |
|  | Choi | Low | Continuous | P = 0.002 \| np | + | x |
|  | Park | High | Continuous  Groups (<4.2 versus ≥4.2) | np \| P < 0.001  P = 0.076 \| P = 0.039 | x  + | +  + |
|  | Tomaszewski | Low | Continuous | np \| P > 0.05 | x | o |
|  | Zhong | Low | Groups (3.25-3.49 versus ≥ 3.5) | P = 0.105 \| P = 0.117 | o | o |
|  | Tauchi | High | Continuous | np \| P = 0.159 | x | o |
| Caliper measured PD severity | Alaca | Low | Groups (mild, moderate, severe) | P < 0.001 \| np | + | x |
| Pectus asymmetry | Hong | Low | Δ chest cavity vertical distance (groups: <10 versus >10mm) | P = 0.001 \| np | + | x |
| (asymmetry vs symmetry) | Choi | Low | Asymmetry index | P = 0.155 \| np | o | x |
|  | Tomaszewski | Low | Δ chest cavity vertical distance (Groups: <10 versus >10mm) | Np | - | x |
|  | Zhong | Low | Symmetric index (groups 0.95 < - < 1.05 versus ≥ 1.05)  Offset coefficient (groups: ≤ 10 versus >10mm) | P = 0.118 \| p > 0.05  P = 1.000 \| P = 0 813 | o  o | o  o |
|  | Park | High | Not described | P = 0.061 \| P = 0.004^*^ | o | + |
| Sternal Tilt Angle | Hong | Low | Continuous | P = 0.019 \| P = 0.294 | + | o |
|  | Choi | Low | Continuous | P = 0.390 \| np | o | x |
|  | Zhong | Low | Groups (<25° versus ≥ 25°) | P = 0.251 \| p > 0.05 | o | o |
|  | Tauchi | High | Continuous | Np \| P = 0.371 | x | o |
| Angle of Louis | Choi | Low | Continuous | P = 0.612 \| np | o | x |
| Flatness index | Choi | Low | Continuous | P= 0.973 \| np | o | x |
| Number of inserted bars during Nuss | Park | High | Single versus multiple | P=0.035 \| P=0.012 | + | + |
| Family history | Waters | Low | Pectus, scoliosis, both | np | o | x |

* value based on population that also included patients with CA<10º.
o, No correlation/no relationship found between predictive factor and AIS
+, Positive association found between predictive factor and AIS
−, Negative association found between predictive factor and AIS
x, Association between predictive factor and AIS was not assessed
BMI = Body Mass Index, na = not applicable, np = not performed.
