# Supplementary material for: Clinical significance of concomitant pectus deformity and adolescent idiopathic scoliosis: systematic review with best evidence synthesis
Source: N Am Spine Soc J. 2022 Jun 25;11:100140. doi: 10.1016/j.xnsj.2022.100140 (PMC9256832; doi:10.1016/j.xnsj.2022.100140)
Supplement: Supplementary file 5 [file mmc5.docx]

Appendix E. Determinants studied as predictors for the transformation of spine curvature after pectus correction.

| Reported determinant | References | Study quality | Measurement method | Statistical analysis  (prevalence \| CA) | Association with postoperative prevalence of scoliosis | Association with decrease of CA | Association with incraese of CA |
| --- | --- | --- | --- | --- | --- | --- | --- |
| Age at pectus correction (in years) | Park | High | Continuous  Groups (<10y versus ≥10y) | Np \| P < 0.001  P *<* 0.001 \| P *<* 0.001 | x  + | +  + | - - |
|  | Chung | High | Continuous | Np \| P = 0.420 | x | o | o |
|  | Iscan | Low | Continuous  - children (<18) - adults (≥18)  Groups (children vs adults) | P = 0.250 \| P = 0.046* P = 0.500 \| P = 0.113*  P = 0.243 \| P = 0.227* | o  o  o | -  o  o | +  o  o |
| Gender | Park | High | Dichotomous | P = 0.003 \| P = 0.048 | + | + | x |
| (female vs | Chung | High | Dichotomous | Np \| P = 0.798 | x | o | o |
| male) | Iscan | Low | Dichotomous | Np \| P = 0.546 | x | o | o |
| Heigth | Park | High | Continuous | Np \| P < 0.001 | x | - | + |
| Weight | Park | High | Continuous | Np \| P < 0.001 | x | - | + |
| BMI | Park | High | Continuous | Np \| P = 0.940 | x | o | x |
| AIS convexity | Chung | High | Right vs left | Np \| P = 0.302 | x | o | o |
| Preoperative Cobb Angle | Park | High | Continuous | Np \| P = 0.090 | x | o | x |
|  | Ghionzoli | Low | Continuous | Np \| P = 0.0011* | x | + | x |
|  | Chung | High | Continuous  Groups (≤15º vs > 15º) | Np \| P < 0.001  Np \| P < 0.001 | x  x | -  - | +  + |
|  | Iscan | Low | Continuous | P = 0.063 \| P = 0.010* | o | - | + |
| Preoperative Haller Index | Chung | High | Continuous | np \| P = 0.125 | x | o | o |
|  | Iscan | Low | Continuous - Mild (<3.2) - Moderate (≥3.2 - < 3.5) - Severe (≥3.5) Groups (mild, moderate, severe) | np \| P = 0.282 P = 1.000 \| P = 0.182 P = 0.250 \| P = 0.049 P = 0.753 \| P = 0.916 | x o  o  o | o  o  -  o | o  o  +  o |
| Preoperative Sternal Tilt Angle | Chung | High | Continuous | np \| P = 0.766 | x | o | o |
|  | Iscan | Low | Continuous - Symmetrical (<5) - Mild (5-14) - Severe (≥15) Groups (symmetrical, mild, severe) | P = 1.000 \| P = 0.067 P = 0.500 \| P = 0.022 P = 0.250 \| P = 0.317  P = 0.081 \| P = 0.865 | o o o  o | o - o  o | o + o  o |
| Preoperative Asymmetry index | Iscan | Low | Continuous - normal (0.05 - +0.05)  - severe (<0.05 or >+0.05)  Groups (normal vs severe) | P = 1.000 \| P = 0.065  P = 0.125 \| P = 0.070  P = 0.940 \| P = 0.546 | o  o  o | o  o  o | o  o  o |
| Number of inserted bars during Nuss | Park | High | Single vs multiple bars | np \| P = 0.002 | x | + | x |
|  | Chung | High | Multiple bars | np \| P = 1.00 | x | o | o |
| Period of bar implementation | Chung | High | Continuous | np \| P = 0.339 | x | o | o |

* value based on population that also included patients with CA<10º.
o, No correlation/no relationship found between predictive factor and postoperative change in curvature
+, Positive association found between predictive factor and postoperative change in curvature
−, Negative association found between predictive factor and postoperative change in curvature
x, Association between predictive factor and postoperative curvature improvement/aggravation was not assessed

BMI = Body Mass Index, np = not performed, vs = versus.
